# Insights Into the Role of Epigenetic Factors Determining the Estrogen Response in Estrogen-Positive Ovarian Cancer and Prospects of Combining Epi-Drugs With Endocrine Therapy

**DOI:** 10.3389/fgene.2022.812077

**Published:** 2022-07-08

**Authors:** Sadia Sarwar, Abir Alamro, Fazlul Huq, Amani Alghamdi

**Affiliations:** ^1^ Riphah Institute of Pharmaceutical Sciences, Riphah International University, Islamabad, Pakistan; ^2^ Department of Biochemistry, College of Science, King Saud University, Riyadh, Saudi Arabia; ^3^ Eman Research Journal, Eman Research, Sydney, NSW, Australia

**Keywords:** ovarian cancer, antiestrogens, chemohormonal, estrogen receptor, epi-drugs, hormone therapy, combination therapy, biomarkers

## Abstract

Ovarian cancer is one of the most lethal malignancies. The population at the risk is continually on the rise due to the acquired drug resistance, high relapse rate, incomplete knowledge of the etiology, cross-talk with other gynecological malignancies, and diagnosis at an advanced stage. Most ovarian tumors are thought to arise in surface epithelium somehow in response to changes in the hormonal environment. Prolonged treatment with hormone replacement therapy (HRT) is also considered a contributing factor. Estrogens influence the etiology and progression of the endocrine/hormone-responsive cancers in a patient-specific manner. The concept of hormonal manipulations got attention during the last half of the 20th century when tamoxifen was approved by the FDA as the first selective estrogen receptor modulator (SERM). Endocrine therapy that has been found to be effective against breast cancer can be an option for ovarian cancer. It is now established that global changes in the epigenetic landscape are not only the hallmark of tumor development but also contribute to the development of resistance to hormone therapy. A set of functionally related genes involved in epigenetic reprogramming are controlled by specific transcription factors (TFs). Thus, the activities of TFs mediate important mechanisms through which epigenetic enzymes and co-factors modify chromatin for the worst outcome in a site-specific manner. Furthermore, the role of epigenetic aberrations involving histone modifications is established in ovarian cancer pathogenesis. This review aims to provide insights on the role of key epigenetic determinants of response as well as resistance to the hormone therapy, the current status of research along with its limitations, and future prospects of epigenetic agents as biomarkers in early diagnosis, prognosis, and personalized treatment strategies. Finally, the possibility of small phytoestrogenic molecules in combination with immunotherapy and epi-drugs targeting ovarian cancer has been discussed.

## Introduction

Preclinical and clinical data suggest an important role of estrogen in the institution and progression of certain ovarian cancers (Langdon et al., 1994; Simpkins et al., 2013). Changes in the size, shape, and histology of ovaries can occur during the menstrual cycle, pregnancy, menopause, and post-menopause. The development of ovarian cancers is linked to ups and downs in hormonal levels during different stages of life. It is also influenced by changes taking place during pregnancy and ovulation with the chances of disease coincidently rising with rising estrogen levels in circulation (Rodriguez et al., 2001; Riman et al., 2002). The Million Women Study and Women’s Health Initiative have also linked the rise in risk with Hormone Replacement Therapy (HRT) (Glud et al., 2004; Beral, 2007). Several other studies have also associated an increased risk of ovarian cancer with HRT. Several reports indicate that incessant ovulation in case of null or low parity has been linked with increased risk while suppression of ovulation (pregnancy, lactation, oral contraceptives) is associated with decreased risk (Falhalla, 1971). According to the histopathological variations, ovarian cancer can be divided into subtypes including epithelial, germ cell, and sex cord-stromal tumors. Among these, up to 90% of malignant ovarian tumors are epithelial, which further can be divided into five subtypes, endometrioid (10%), mucinous (3%), clear cell (10%), low-grade serous (<5%), and high-grade serous (70%) (Peres et al., 2018). Low-grade serous ovarian cancer is associated with the higher expression of estrogen receptors (Hunter et al., 2015). However, there are some other inconsistent reports reporting the expression status of ERα to be expressed in 80% of high-grade serous ovarian cancer (Halon et al., 2011; Matsuo et al., 2014). The application of endocrine therapy in the case of breast and endometrial cancers has made it an emerging research direction for a targeted cure of ovarian cancer (Onwude 2015). According to the guidelines of NCCN OC and that of the European Society of Gynecological Oncology, endocrine therapy may be used to treat patients with platinum-resistant and recurrent ovarian cancer (Daly et al., 2020; Colombo et al., 2021). It is now well established that epigenetic factors are as important as are genetic factors in regulating the cancer biology, driving the primary tumor growth and invasion, and modulating the immune response within the tumor microenvironment (Roulois et al., 2015; Deblois et al., 2020). Thanks to high-throughput CRISPR-Cas9 dropout screening, which has enabled researchers through functional genomic screenings to find out the specific driver or fitness genes that are differentially expressed in tumor tissues including ovarian ones and are essentially perturbed in cancer cells under a specific environment (Faber et al., 2013; Yu and Yusa, 2019). Alexandrova et al. (2020) performed Gene Ontology analysis and elucidated functional pathways linked to the fitness genes in the ovarian cancer context and obtained a distribution map of the fitness genes which were key hallmarks of cancer, including estrogen-mediated S-phase entry (cell cycle regulation) and estrogen receptor signaling (for detailed study, please see the article). They identified that 35 ERα associated fitness genes are linked to many pathways including cell cycle regulation and cell proliferation (MTOR, CCND1 CDK2 CCNA2 CDK, PCNA, PPP1R12A PIK3C3 EIF4E HDAC3, and PPP1CB), immune response activation (EIF2B3, EIF2B4, EIF2B2, and EIF2B5), and RNA polymerase II-dependent transcription (Moldovan et al., 2007). The same group further explored three Ovarian Serous Cystadenocarcinoma cohorts comprising 1,680 ovarian cancer tissues from 1,668 patients and found that estrogen receptor pathway associated fitness genes in ovarian cancer cells were altered, mostly amplified, in 74% of patient tissues. Targeting the dysregulated epigenetic avenues is less challenging, and it is easier to target by small molecule drugs than fixing genetic mutations. It has also been established that epigenome modulations sensitize solid tumors toward immunotherapy. Cancer cells evade the immune system by epigenetically silencing the expression of cell surface molecules that render them vulnerable to immune targeting. De-repression of tumor-associated antigens with epi-drugs may render cancer cells and tumors susceptible to elimination by the immune system (Garcia-Martinez et al., 2021). The overall outcome of these developments is the increased interest in epigenetics-based diagnostic and prognostic tools. Notable are the DNA methylation diagnostic screens either in the development phase or in clinical trials (Berdasco and Esteller, 2019). Epigenetic alterations not only contribute to tumor progression but also to the acquisition of chemo-resistance (Zeller et al., 2012; Lue et al., 2015). The development of epi-drugs is an important development in precision medicine. Currently, many epi-drugs are under clinical trials including inhibitors of methyltransferases and histone deacetylases while nine such agents have been approved by FDA. Among these trials, breast cancer phase II trials (NCT04190056, NCT00828854, and NCT00676663) are of interest which is testing the efficiency of epi-drugs in combination with traditional therapies (Garcia-Martinez et al., 2021). The most promising chromatin-modifying agents belong to histone de-acetyltransferases inhibitors (HDACs). The epi-drugs of this class are in different phases of clinical trials for the treatment of ovarian cancer (Yang et al., 2018). Only three among those devise personalized treatment strategies and provide better care to patients with ER + ovarian cancer.

### Estrogen Receptors and Downstream Signaling Pathways

Estrogen receptors (ERα and ERβ) are steroidal hormone receptors (nuclear) that belong to the nuclear receptor superfamily of transcription factors, along with others including androgen receptors, thyroid receptors, progesterone receptors, vitamin D receptors, retinoic acid receptors, and mineralo-corticoid receptor (Pearce and Jordan., 2004). Both are involved in the transcription of target genes on binding to endogenous and exogenous ligands, thus mediating reproductive organ development and function, as well as pathological processes (breast, ovarian and endometrial cancer). Alongside, many other interlinked pathways and proteins are also affected in a very complicated way. However, other receptors (previously categorized as orphan receptors) located in the plasma membrane and endoplasmic reticulum known as G protein-coupled estrogen receptors (GPER) are the focus of recent research. Rapid cellular responses initially referred to as “nongenomic” lately have been found to be induced through G protein-coupled estrogen receptor (GPER). The role of GPER in exerting nongenomic estrogen effects is a topic of high interest. GPER is reported to be highly expressed in cancerous breast tissue as compared to normal tissue. In estrogen-responsive breast tumors, GPER expression has been found to be associated with poor prognosis and low survival rate (Filardo et al., 2008; Martin et al., 2018). In response to E2, the GPER signaling pathway acts through the generation of certain messengers (Ca2, NO, and cAMP) and activation of receptor tyrosine kinases MAPKs, EGFR, PI3K/Akt, and SRC. It is well known that GPER activates EGFR which increases the expression of matrix metalloproteinase (MMP) expression and activates downstream Src-related tyrosine kinase family (Filardo et al., 2008). These events activate the MAPK/PI3K pathways. In ER + breast cancer cells (MCF-7), GPER and downstream PI3K/MAPK/STAT pathways were shown to be mediating apoptosis as well as resistance in response to tamoxifen (Rouhimoghadam et al., 2018). Activation of EGFR also triggers the downstream Src kinase family to phosphorylate Raf, which in turn stimulates extracellular signal-regulated kinases (ERK1/2) phosphorylation, and activation of a number of transcription factors including, c-Myc, c-fos, and c-jun (McCubrey et al., 2007). Low-grade serous ovarian cancer seems to be mediated through activation of the MAPK pathway *via* RAS/RAF and is associated with high levels of estrogen receptor and progesterone receptor expression (Hunter et al., 2015). However, in another report, the ovarian cancer cells SKOV3 (ER responsive) were reported to undergo apoptosis through cell cycle arrest, which was mediated through GPER (Ignatov et al., 2013). While the association of GPER with many other neighboring pathways is now well supported by literature evidence (some of which mentioned above), the inconsistent observations regarding the final outcome seem to be influenced by the specificity of ligands and cell or tissue type which decides whether GPER may result in the suppression or progression of cancerous cells.

### Interactions of Estrogens and Antiestrogens With Estrogen Receptors

**FIGURE 1:**
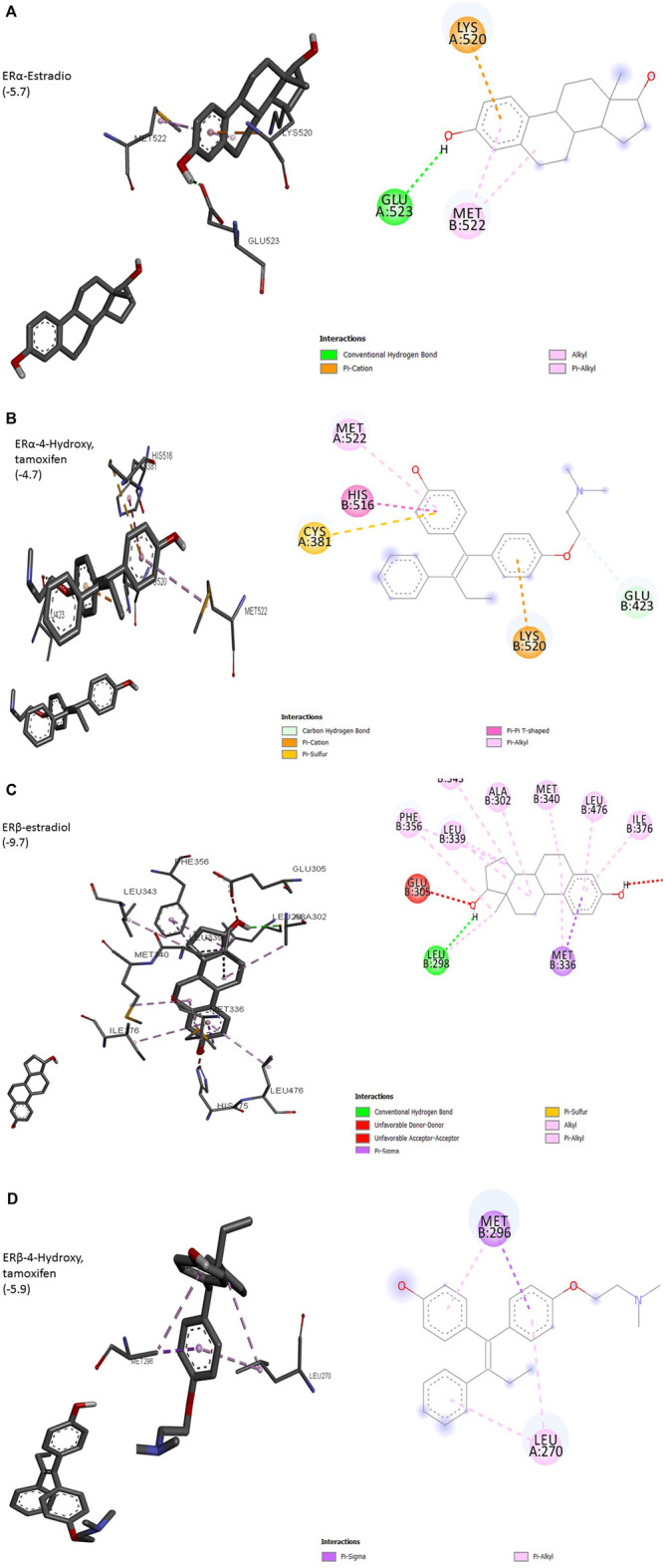
(Continued).

The interaction of a particular ligand depends on the tissue or organ (or distinct cellular subtypes within a tissue or organ) due to different relative abundances of specific receptors in that tissue or organ. Several studies have reported the presence of both ERs in the breast, brain, bone, urogenital tract, ovaries, and cardiovascular system (Kuiper et al., 1997). ERα is dominant in the uterus and ovary in cancerous while ERβ is predominant in normal ovaries. Different effects arising from the activation of ERα or ERβ by different ligands seem to arise due to the recruitment of different co-activators. Most of the studies on ligand–receptor interactions have been carried out using ERα as a model. The size of the ligand-binding site of ER (450Å) is much larger than the size of its natural ligand estradiol (250Å). The binding of estradiol involves a hydrogen bond between hydroxyl groups of A ring to Glu523 (Figure 1A). The bulk of ligand (E2) involving Rings A and C undergoes hydrophobic interactions with Met522 and Lys520. This arrangement exposes AF-2 to be available to co-activators, a requirement for the transcription process to be initiated. ERα complexed with antagonists 4-OH tamoxifen has shown hydrogen bonding between hydroxyl groups at A ring with Glu423 (Figure 1B). Overall, these changes result in adjusting the position of AF-2 in such a way that co-repressor is recruited instead of a co-activator. 3D structure of other isoforms, ERβ, has shown that its ligand-binding domain is very similar to that of ERα but still the differential expression of two isoforms in different organs is evident. Preferences for ligands are also obvious, especially towards phytoestrogens. The size of the binding domain of ERβ is smaller than that of ERα due to the replacement of smaller Glu523 with larger and bulky Leu298 in ERβ (Figures 1A,C). Almost all-important known phytoestrogens including genistein, coumestrol, and apigenin are found to have a greater affinity for ERβ. The main obvious reason for this preference is the above substitution of amino acids. The hydroxyl groups of genistein appear to contribute to this preference because the loss of one (daidzein) or both hydroxyl groups (formononetin) results in a reduced preference for ERβ. However, the antagonist 4-hydroxy tamoxifen does not show any favorable interaction with ERβ (Figure 1D). This differential cellular response to the compounds of different origins depends upon the pattern of ERα/ERβ expression and their specific preferences for ligands (Kuiper et al., 1998; Pike et al., 1999).

### Interactions of Epigenetic Landscape With Estrogen Signaling

**FIGURE 2:**
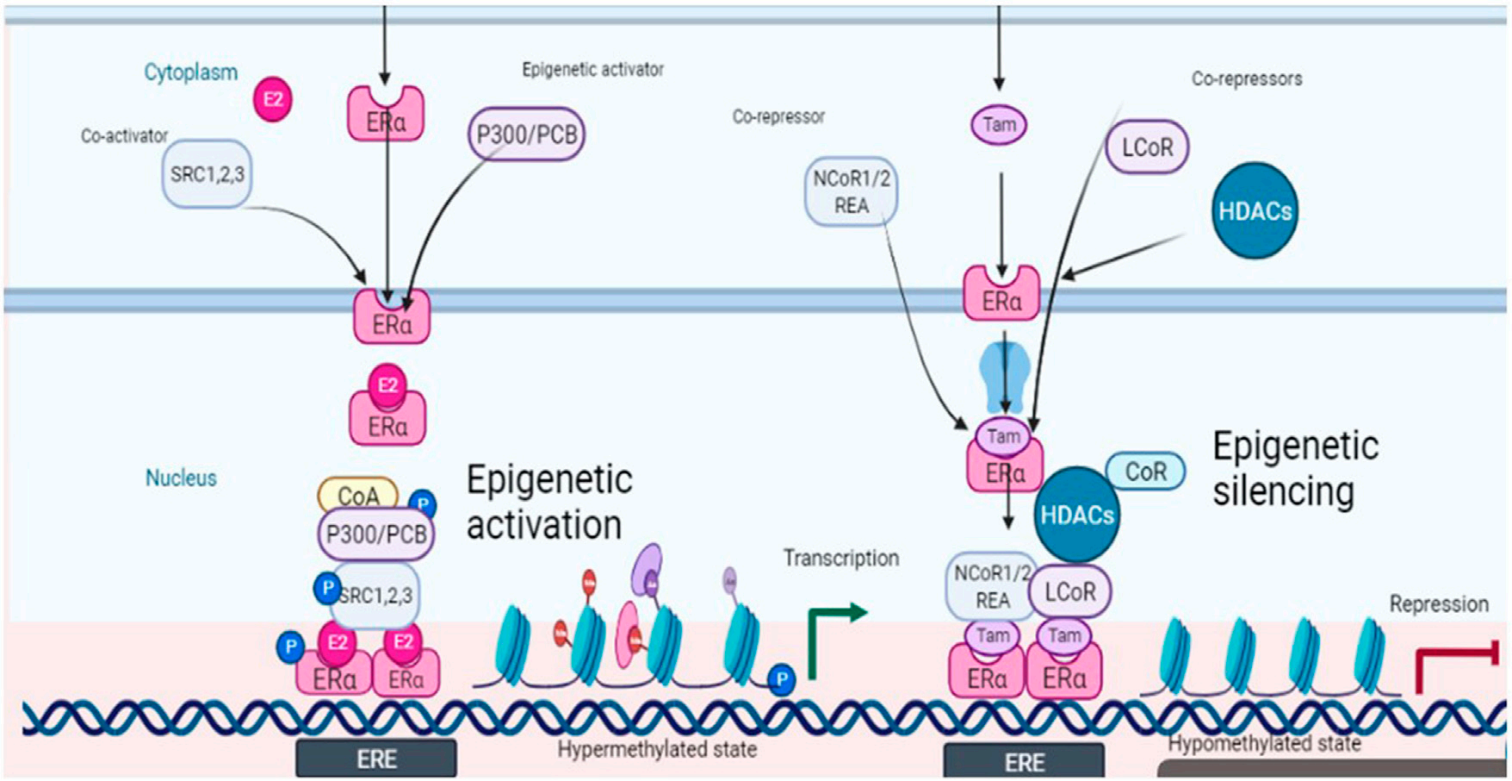
Schematic depiction of estrogen signaling and its interactions with epigenetic events in response to estradiol (ligand) and tamoxifen (antagonist).

The prospects of epigenetic control are being rapidly explored because of the possibility of reversing the malignant events, contrary to the irreversible genetic events. The epigenetic landscape is strongly influenced by histone modifications mainly through DNA methylation and histone acetylation. Among eight histone modifications (phosphorylation, ubiquitination, glycosylation, ribosylation, carbonylation, sumoylation, ADP-ribosylation, methylation, and acetylation), disturbed acetylation and methylation manifest themselves as the genesis of cancer including ovarian tumors (Huang et al., 2016). Histone acetyltransferases (HATS), histone de-acetyltransferases (HDACs), histone methyltransferases (HMTs), and histone demethylases (HDMTs) control the acetylation and methylation of histone proteins of chromatin nucleosome. The steady-state of chromatin is maintained as long as the balance between histone methyl transferases-demethylases and acetyltransferases-deacetylases is maintained, in a normal cell. One manifestation of the disturbed balance towards hyper-methylation and increased histone deacetylation is cancer including ovarian malignancies (Yang et al., 2018). The epigenome is a fundamental determinant controlling the outcome of endocrine therapy (estrogen-based) in breast cancer. The development of epigenetic targeting therapeutics is based on understanding the complex molecular language of chromatin signaling and its interactions with estrogen signaling. Activation functions (AF) are important in this context. They act as guides in the recruitment of co-activators (promoters or repressors) which on deployment to AF result in the initiation or inhibition of transcription. This has been explained in Figure 2 with reference to the breast cancer in response to clinically used selective estrogen receptor modulator (SERM) tamoxifen, the first recognized endocrine therapeutic agent. These events are cell specific. Accordingly, cells sense these ER-ligand interactions and coordinate the regulation of gene transcription. AF1 is a common phosphorylation target of mitogenic kinases to alter ERα transcriptional activity. ERα dimerization occurs on the LBD interface, which also binds E2, resulting in a conformational shift. AF2, the major transcriptional activation domain, mediates co-regulator interactions based on this confirmation. The binding of agonists with estrogen receptors results in their dimerization and subsequent interaction with estrogen response elements ERE (DNA sequence specific for ERα) in the promoters of target genes. As explained in Figure 2, some co-activators are also activated (recruited) in response to this ER-ERE complex formation. The role of SRC-1, SRC-2, and SRC-3 (steroid receptor co-activators types) are among the co-activators that directly interact with DNA besides indirectly recruiting some other integrators including p300/CREB-binding protein (CBP/CREBBP). P300/CBP is one of the two histone acetyltransferases (HATs), a key controlling factor of epigenetic events. In ER + cancers, FOXA1, GATA3, PBX1, and AP-2γ bind specific DNA target sequences in condensed chromatin and facilitate ERα chromatin binding in response to E2 stimulation through deploying activating epigenetic marks such as H4R3me1 and H3K27Ac (resulting in hyper-methylation and acetylation). Activated ERα can also recruit a cohort of co-activators or co-repressors to mediate gene transcription or repression blocking the gene transcription (co-activators amplify transcription through attachments of ERs onto variable sites but have specific sites for interaction with the receptor) (Garcia-Martinez et al., 2021). One such co-activator of established repute in the case of breast cancer is histone methyltransferase DOT1L, which has been suggested to be of prognostic value in ovarian cancer also. It is a promoter of cell cycle progression and drug resistance by acting as a transcriptional co-regulator (Zhang et al., 2017) and as a co-factor acting on multi-drug resistance genes (Liu et al., 2018). However, recent evidence suggests that inhibition of estrogen signaling by targeting this upstream ERα co-factor and regulator may be an effective therapeutic approach towards ERα positive ovarian cancers. ERα and DOT1L co-expression is an indicator of poor survival in ovarian cancer patients. Interfering with his duet with ERα/DOT1L inhibition with selective antagonists has been shown to result in the reduction of OC cell proliferation, a G1 phase arrest, mediated by significant changes in the cell transcriptome. It might be the outcome of selective inhibition of either protein interfering with ERα and DOT1L co-recruitment and H3K79 methylation (Salvati et al., 2019).

Another important factor in this context is histone acetylation and deacetylation associated with gene transcription and repression respectively. Both co-activators and co-repressors also have acetyltransferase activity contributing to both histone acetylation and histone deacetylation. The former is associated with transcription while the latter is associated with repression. A co-repressor of estrogen receptor activity (REA) was also isolated in 1999 which later on was shown to selectively enhance the effectiveness of antiestrogens towards estrogen receptors as compared to un-liganded ER (Montano et al., 1999). REA contributes more towards selective action by competing with SRC-1 towards the trans-activating transcriptional activity of ER. Hence the elevated level of REA in the cells is a factor favoring the antiestrogenic action (Katzenellenbogan et al., 2000). Besides ligands, co-activators also have specific preferences as applied to the activation role. An example of such a co-activator is p68 RNA helicase which is more specific towards AF1 on ERα and favors tamoxifen-ERα induced transcription (Endoh et al., 1999). However, through interactions with co-repressors such as LCOR and NCoR1/2, liganded ERα and tamoxifen-bound ERα also recruit epigenetic repressors including histone deacetylases (HDACs) and the NuRD complex to mediate gene repression by removing active epigenetic marks (Figure 2).

Now it is recognized that global chromatin organization, specific spatial arrangement of chromosomes in the nucleus, is critical in genome stability and gene regulation. The transcription of genes can be affected or modulated through the repositioning of the chromatin. It was revealed *in vitro* in MCF-7 cells using high-throughput chromosome conformation capture (Hi-C) study that hormones and estrogens can modulate or influence chromatin organization by changing the activating and repressive chromosomal marks. In response to ERα binding, gene expression was modulated through higher spatial compartmentalization of active and repressed marks which interacted differentially in response to E2 induction (Mourad et al., 2014). More enriched loci were related to cancer progression. ERα was revealed as a chromatin interaction organizer. However, such studies are needed in ovarian cancer cells or tissues to better relate this phenomenon in this context.

### Current Status of Endocrine Therapy as Combination Therapy

Increased interest in hormone manipulations in gynecological cancers resulted in significant new information. It is well recognized that at physiological concentrations, estrogens can also regulate some important signaling pathways including cAMP, PKC, and MAPK. Once activated, MAPK can be a key player in ER-mediated signaling. Induced activation may involve ERK1, 2, 3, 5, MEKs, and tyrosine kinases in ras/raf dependent or independent mode. In the absence of estrogenic stimulus, several mitogenic growth factors can induce estrogenic effects in ER-positive cells; especially growth factor receptor tyrosine kinases can activate MAPKs. MAPK phosphorylates ER at ser followed by the transcription, thus inducing a ligand-independent activation of ER. Such ligand-independent activation of ER produces a weak estrogenic effect because it does not involve all estrogen-regulated genes (Suga et al., 2007). There have been several reports from *in vitro* studies showing that antiestrogens can block such ligand-independent activity. Inhibitory effects of antiestrogens may result from their interactions with the MAPK pathway. Several other reports have shown that antiestrogens or aromatase inhibitors cause estrogen deprivation resulting in cell cycle arrest at the G1 phase by inducing p53, p21, and p27 inhibitors of cyclin-dependent kinases in the breast as well as ovarian cancers (Cariou et al., 2000). This results from indirect inhibition of cell proliferation by interfering with MAPK/ERK. However, this kind of activation through MAPK, independent of ER signaling, may contribute to antiestrogen resistance. These observations strengthen the hypothesis that blocking such interlinked pathways may be effective in achieving desired therapeutic outcomes. In one study, the combination of antiestrogen fulvestrant (AI) with MEK inhibitor decreased ovarian cancer cells growth through enhanced p53 and p21 thus providing support to the said hypothesis (Slomovitz et al., 2020). In a recent preclinical investigation reversal of fulvestrant resistance with Src inhibition in ER + ovarian cancer cell line and xenograft tumor model was observed (Simpkins et al., 2013). These and many other studies explain the presence of many rapid responses which are unlikely to arise from the transcription route. Such cell-specific regulations can be seen to result from the interplay of several epigenetic factors including co-regulator/co-repressor recruitment, ligands, cellular environment, the status of phosphorylation of ER and estrogen pathway, and homo or hetero-dimerization of receptors. This interplay is being manipulated in three phases II clinical trials (NCT04190056, NCT00828854, and NCT00676663) through the combination of endocrine therapy/chemotherapy with epi-drugs in hormone-responsive breast (Garcia-Martinez et al., 2021).

### Future Prospects of Endocrine Therapy in Ovarian Cancer

Looking back to the 1970s, when hormone/endocrine therapy was not considered to be a realistic option, there has been a significant advancement in understanding the basic underlying mechanisms. Consequently, adjuvant endocrine therapy using antiestrogens has resulted in increased survival of thousands of breast cancer patients (Jordan et al., 2011). However, the situation is somewhat different in ovarian cancer. In spite of the fact that most ovarian tumors are endocrine responsive and hormone-dependent (as it is the case for normal ovaries), endocrine therapy has less encouraging response. A careful analysis of reported results (as shown in Table 1) employing hormone therapy, alone as well as in combination, indicates multiple deficiencies in the conducted studies including lack of systematic trials, lack of verification of ER expression, inconsistencies in the correlation of response with ER status, resistance to antiestrogens, inadequate treatment design and lack of placebo versus hormone/chemo-hormonal therapy. It is only recently that a better understanding of the proper patient selection and sensitivity of specific tumor subtypes to endocrine therapy has given some insight as to what is needed to be done to better exploit its potential. Inappropriate patient selection for any treatment regime can worsen the efficacy while the same therapy if applied to proper targets may markedly improve the response. Almost more than 90% of the available reports on clinical studies employing hormone therapy are based on tamoxifen (SERM) and letrozole (AI). In a review of the results of hormone therapy in ovarian cancer reported up to that date, Simpkins et al., 2013 discussed nineteen trials of tamoxifen (SERM), as monotherapy, in recurrent ovarian cancer patients over the last three decades. Out of the total of 695 recurrent patients, the overall response rate was 13%; complete response was 4% and partial response was 9%; disease stabilization was 35% (mean response duration 2–24 months; mean survival 6–36 months) while the progressive disease was 36%. Estrogen receptor status was considered only in 6 out of 19 reports (out of those where ER status was stated 53% were ER+). On the basis of this analysis, they recommended the use of estrogen antagonism for patients who did not receive prior heavy treatment or who have an asymptomatic recurrence, or who need a break from regular chemotherapy because of poor tolerance. Evidence of the use of third-generation aromatase inhibitor letrozole is a mix (Table1) (Bowman et al., 2002; Papadimititrou et al., 2004; Kavanagh et al., 2007). Better clinical benefit in terms of complete and partial response was obtained in response to letrozole in two small to medium scale studies when estrogen receptor status (positive) was a criterion of patient selection. Langdon et al., 2020 in a review on the use of third-generation aromatase inhibitor letrozole strongly advocated the use of letrozole in epithelial ovarian cancer patients with recurrent disease. In another investigation, they found a direct association between CA125 response and ERα expression indicated by an increase in response rate with the increase in expression of ERα.The work provided support to the use of letrozole as adjuvant therapy to prolong the progression-free survival following surgery/chemotherapy when the residual disease is minimal. In other studies investigating the role of two other 3rd generation aromatase inhibitors, anastrozole, and exemestane, disease stabilization was significant (DelCarmen et al., 2003; Freedman et al., 2006). Careful analysis of conducted clinical trials reveals that results are more positive in well-planned and well-conducted studies and a combination of hormonal agents with chemotherapy (cisplatin in this case) showed a better response than monotherapy (Table1). In this regard, the selection of proper patients is a crucial factor. In a clinical trial including both platinum-sensitive and platinum-resistant patients, 64% of the former group and 39% of the latter group responded positively to the combination of cisplatin with tamoxifen while the overall response rate was 50% (30% complete; 20% partial), overall median survival was 23 and 19 months with mild to moderate toxicities (Panici et al., 2001). A very important study highlights specific circumstances where tamoxifen can be the treatment of choice. It has been shown that tamoxifen is a highly favorable agent compared to cytotoxic chemotherapy in recurrent ovarian cancer patients with chronic kidney disease (Sirisabya et al., 2008). Whereas traditional chemotherapy may aggravate the condition due to additional toxicity, no major adverse reactions (hot flashes and nausea) occurred when tamoxifen was used (20 mg/day) in a group of 29 ovarian cancer patients (22 epithelial; 4 peritoneal and 3 fallopian tube). Thirteen showed mild CKD, thirteen moderate, and the remaining three severe CKD. The response was complete (0–18%), partial (10%), disease stabilization (5–83%), and disease progression (48%) respectively. Small sample size, and inconsistencies in treatment and response evaluations, however, were the limitations of this study. Thus, large-scale studies are needed to better understand pharmacokinetics.

**TABLE 1:** Response to the use of antiestrogens and aromatase inhibitors in recurrent ovarian cancer patients administered alone and in combination in the literature.

Agent	Dose	Number of patients	ER status	Recurrent/resistant to chemotherapy	Complete response	Partial response	Stable disease
Tamoxifen	20 mg BID	29	ND	Yes	3%	7%	21%
Tamoxifen	20 mg/day	1	ER+	Yes	-	-	Yes
Tamoxifen	20 mg	29	ND	Yes	0%	10%	41%
Tamoxifen	20 mg	105	62/105 ER+	Yes	10%	8%	38%
Tamoxifen	20 mg	102	ND	Yes	10%	7%	
Tamoxifen	20 mg	13	ER+/ER-	Yes	0%	8%	31%
Tamoxifen	Initially 40 mg/day for a week followed by 20 mg/day	31	4/11 ER+/ER	Yes	3%	6%	19%
Fulvestrant (pure antagonist)	1000 mg on day 1 followed by 500 mg intermittently on 14th, 28th and every 28 days hereafter	26	ER+	Yes	4%	4%	35%
Letrozole	2.5 mg/day	33	33/33 ER+	Yes	0%	3%	21%
Letrozole	2.5 mg/day	13	13/13 ER+	Yes	15%	15%	39%
Letrozole	2.5 mg/day	27	20/27 ER+	Yes	14%	11%	18%
Letrozole	2.5 mg/day	42	42/42 ER+	Yes			Overall response >50%
Letrozole	2.5 mg/day	54	ER+/ER-	Yes	0%	9%	26%
Anastrazole	1 mg/day	53	ND	Yes			44% clinical benefit
Exemestane	25 mg/day	22	ER+16/22	Yes	0%	0%	36%
Tamoxifen + goserelin (Hormonal agent)	Tamoxifen 20 mg twice/day; Goserelin 3.6 mg/month	26	ER+	Yes	3.8%	7.7%	38.5%
Tamoxifen + cisplatin	80 mg/day for 30 days followed by 40 mg/day	50	ER+/ER-	Yes			

### Areas Needed to be Focussed for Better Outcome

The need to develop proper biomarkers to identify patients with tumors sensitive to endocrine therapy is obvious. For proper patient selection, there is a need for early-stage detection of expression profiles of sensitive tumors which are different from insensitive ones. Proper assessment of the association of the biomarkers with clinical outcomes is also highly desirable. While some of the target genes induced by ERα are found to be expressed in both breast and ovarian cancers, some others are exclusive to ovarian cancer. In breast cancer, PR, cathepsin D, C-FOS, and PS2 have been identified to be significant predictors of response to antiestrogen therapy (Munez et al., 1987; Jordan et al., 2001; Rochefort et al., 2003). PR, cathepsin D, and C-FOS have also been detected in ER + ovarian cancers while C-MYC, SDF-1, and TGFα have been found to be exclusively expressed in ovarian tumors. Fibulin-1C and cyclins (D1, A, and E) were shown to be related to ovarian cancer cell invasion and cell cycle regulation respectively (Chien et al., 1994; Clinton and Hua, 1997; Rochefort et al., 2003; Albanito et al., 2007). In an analysis of 1200 cancer-related genes in ER + ovarian cancer cell lines, PEO1, TNFDF1, FOSL1, TRAP1, cathepsin D, and TFAP4 were found to be upregulated in response to insult with estrogens (O'Donnell et al., 2005; Chien et al., 1999; Hall and Korach, 2003). The results of two studies published in 2007 confirmed the presence of some already known estrogen-regulated proteins along with some new ones (Table 2) responsive to letrozole (Walker et al., 2007a; Walker et al., 2007b). Among them, insulin-like growth factor binding protein4 (IGFBP4), trefoil factors (TFF1 and TFF3), TNF receptor-associated protein1, topoisomerase II alpha (TOP2A), and ubiquitin-conjugating enzyme E2C (UBE2C) were found to be upregulated while insulin-like growth factor binding proteins 3 and 5 (IGFBP3, IGFBP5), vimentin, plasminogen activator, urokinase (PLAU) and Beta IG-H3 (BIGH3) were downregulated in response to letrozole (Bowman et al., 2002; Walker et al., 2007b). Another complication is the fact that invasive epithelial ovarian cancer is not a single homogenous disease but is comprised of five distinct subtypes. The five types are not only distinct phenotypically, etiologically, and molecularly but also in the relation between biomarker expression and survival. The occurrence of inconsistencies in histology as well as pathology across these subtypes (except in HGSc) has been the main obstacle to proper biomarker identification. An inspiring report based on a large study on 2933 women was published (Sieh et al., 2013). Progesterone receptor (PR) and ER expression were projected, on the basis of this study, as potential prognostic biomarkers for endometroid and high-grade serous ovarian cancers. It was found that expression of both ER and PR was associated with significant improvement in disease-specific survival as compared to those showing no hormone expression. To use the aforesaid biomarkers as criteria in patient selection for personalized treatment, better clinical studies are needed as are further studies exploring hormone pathways and their association with other pathways.

**TABLE 2:** Biomarkers associated with estrogen/antiestrogen response.

Biomarker	Expression status in responseto letrozole	Response to fulvestrant clinical response	Progression-free survival
ER	Upregulated	Significant	Non-significant
PGR	Upregulated	Non-significant	Non-significant
IGFBP4	Upregulated	Non-significant	Non-significant
TFF1	Upregulated	Non-significant	Significant
TFF3	Upregulated	Non-significant	Non-significant
TRAP1	Upregulated	Non-significant	Non-significant
TOP2A	Upregulated	Non-significant	Non-significant
Vimentin	Downregulated	Significant	Significant
PLAU	Downregulated	Non-significant	Non-significant
IGFBP5	Downregulated	Non-significant	Non-significant

Resistance to antiestrogen therapy in ovarian cancer cell lines is a practical concern and an obstacle. A considerably large proportion of estrogen-responsive ovarian cancers initially responding to even single hormonal agents (antiestrogens) become resistant after only a few months. The resistance is innate in ovarian cancers. Investigation for markers predicting resistance to antiestrogens is equally important. Histone modifications pattern insensitive and resistant ER + ovarian cancers need to be explored to be developed into new therapeutic targets in the case of endocrine-resistant patients. Many *in vitro* combination studies have been carried out using phytoestrogens (resveratrol, genistein, thymoquinone, wedelolactone) in combination with platinum drugs in an effort to find combinations that could overcome/reverse resistance in ovarian cancer cell lines (Sarwar et al., 2021). Among several combinations of phytochemicals with cisplatin and other platinum drugs, some of the combinations have been found to be synergistic. These synergistic combinations can have additional benefits of reduced systemic toxicity and overcoming resistance (Nessa et al., 2011; Nessa et al., 2012). Another important aspect of antiestrogens in the apoptotic function observed even in ER-negative ovarian cancer cell lines as observed in response to tamoxifen and ICI182, 780 (Ercoli et al., 2000; Mabuchi et al., 2004). Tamoxifen was found to bind competitively to type II estrogen binding sites (EBS) in ovarian tumors. While both cisplatin and tamoxifen showed an anti-proliferative effect, their combination was found to be synergistic with 50-fold increase in potential (Scambia et al., 1992). In another study (McClay et al., 1994) a decrease in the rate and magnitude of delay to resistance in ovarian cancer cell lines by a factor of 2.4 (*p* < 0.01) was found to result from concurrent exposure of cancer cells to tamoxifen and cisplatin; delay in the development of resistance to cisplatin due to the combined treatment may have important clinical implications. Similar observations were made in oral squamous cell carcinoma (OSCC) cell lines where treatments using a combination of cisplatin and tamoxifen increased cytotoxic and apoptotic effects (Kim et al., 2005). Few reports were published in the last decade, one of them conducted by the Gynecologic Oncology Group (GOG) using an epidrug belinostat—in combination with carboplatin in resistant ovarian cancer patients which had to be closed due to lack of drug activity (Dizon et al., 2012). However, in the phase II expansion study, they used belinostat in combination with a platinum-based regime comprising carboplatin and paclitaxel offered clinical benefits to epithelial ovarian cancer patients (Dizon et al., 2012). Belinostat is an inhibitor of histone deacetylation (HDACis). There are few other HDACis in Phase I and II or preclinical studies including Trichostatin, valporic acid, romedepsin mocetinostat, and panobinostat. Presently combination of chemotherapy and hormone therapy makes up 5% of total combinations in trials in the case of ovarian cancer. The promising results of the combination of epi-drugs such as HDACs in combination with tamoxifen and CDK4/6 inhibitors (ribociclib) and aromatase inhibitors (exemestan) to restore sensitivity to endocrine sensitivity in estrogen-responsive breast cancer is an encouragement to the researchers and clinicians for such combination regime in estrogen-responsive ovarian cancers.

## Conclusion

In ovarian cancer, the progress in treatment has been quite slow and there is a need to explore all potential avenues towards a better outcome. Antiestrogens have prospects in recurrent and platinum-resistant ovarian cancer as they have shown better response in disease stabilization and occasionally, complete cure. If identified earlier, this approach can be the primary treatment option for specific patients (with tumors sensitive to estrogen blockade or deprivation) with much better chances of recovery if treated early rather than the last option. However, proper patient selection is the key to success. The epigenome is being recognized as a detrimental factor in hormone therapy response; epigenetic factors may also be promising targets for the improved outcome of endocrine therapy in estrogen-responsive ovarian cancer. However, there is much to do in this regard. There is a need to explore and highlight epigenetic mechanisms underlying the pathogenesis of ER + ovarian cancer. This may provide new opportunities for targeting estrogen-induced ovarian cancer with better armament. Regarding diagnosis and prognosis, currently, there are no reliable predictive markers of the disease. Those in clinical use or the preclinical stage suffers from limitations including a lack of sensitivity and selectivity in estrogen-responsive and non-responsive diseases. In this perspective, histone modification status in both ER + and other ovarian cancer can be explored as a biomarker for early diagnosis as well as prognosis. However, much more yet need to be done to explore this approach before it may find a way into clinical care.
